# Effects of Corn–Soybean Meal-Based Fermented Feed Supplementation on Growth Performance, Meat Quality, Fatty Acid Profiles, Nutritional Values, and Gut Microbiota of Lean-Type Finishing Pigs

**DOI:** 10.3390/foods14152641

**Published:** 2025-07-28

**Authors:** Jiao Song, Xin Wang, Yuhan Cao, Yue He, Ye Yang

**Affiliations:** 1 College of Life Science, Yangtze University, Jingzhou 434025, China; songjiao881127@126.com (J.S.); wx11064012@126.com (X.W.); 2 College of Animal Science and Technology, Yangtze University, Jingzhou 434025, China; 15756303545@163.com (Y.C.); 13177112816@163.com (Y.H.)

**Keywords:** fermented feed, lean-type finishing pigs, growth performance, meat quality, nutritional values, fatty acid profiles, gut microbiota

## Abstract

This research investigated the impact of corn–soybean meal-based fermented feed on the growth performance, pork quality, and fatty acid profiles of lean-type finishing pigs. A total of 80 lean-type growing DLY (Duroc × Landrace–Yorkshire) pigs were randomly assigned to 2 groups, with 5 replicates of 8 pigs per pen. The pigs in control group (CON group) were fed a basal diet, while the pigs in fermented feed group (FF group) were fed a diet supplemented with 10% fermented feed. The experimental period lasted 70 days. Results exhibited that pigs in FF group had a significant increase in final body weight and average daily gain (ADG) (*p* < 0.05) and had a significant decrease in the feed-to-gain ratio (F/G) (*p* < 0.05). The FF group also exhibited significant promotion in muscle intramuscular fat content, marbling score, and meat color and significantly reduced the meat shear force and drip loss (*p* < 0.05). Serum analysis indicated that fermented feed significantly elevated blood glucose, total cholesterol, triglyceride levels, and serum hormones such as insulin, leptin, and IGF-1 (*p* < 0.05). Additionally, fermented feed significantly elevated the levels of polyunsaturated fatty acids (PUFAs) and monounsaturated fatty acids (MUFAs), whereas it decreased the saturated fatty acids (SFAs) contents (*p* < 0.05). The fermented feed also significantly enhanced pork nutritional values (*p* < 0.05). The fermented feed increased the expression of *IGF-1*, *SREBP1c*, *PDE3*, *PPARγ*, *SCD5*, and *FAT*/*CD36* mRNA (*p* < 0.05). Furthermore, microbial 16S rDNA analysis uncovered that FF supplementation significantly reduced the *Campilobacterota* phylum abundance, while increasing the genus abundances of *Clostridium_sensu_stricto*, *norank_f_Oscillospiraceae*, *unclassified_c_Clostridia*, and *V9D2013* (*p* < 0.05). In summary, the results indicated that the microbial fermented feed exhibited the regulation effects on pork quality and nutritional values of lean-type pigs through regulating lipid metabolism and gut microbial composition.

## 1. Introduction

Pork is the main animal protein for human health and growth in China. With the enhancement of living standards, people are more concerned about the pork quality and safety. Moreover, the excellent pork quality contributes to enhance the purchase decision of consumers, which is also the critical factor affecting the development of pig husbandry [1]. Therefore, improving the quality and flavor of pork has always been a major concern for consumers and producers. Pork quality is a complicated evaluation index, mainly including physicochemical quality (mainly including meat color, shear force, pH, and drip loss), sensory evaluation attributes (mainly including aroma, tenderness, flavour/taste, and juiciness), and nutritional values [1]. Especially, the sensory evaluation attributes are critical factors in determining the consumer’s acceptability. Though the recent research clarified the pork sensory evaluation attributes using objective evaluation method (e.g., electronic tongue and chromatographic techniques) and subjective evaluation method (e.g., consumer panel sessions) [2,3], the method of pork sensory evaluation attributes is still complex. But the numerous studies have shown that this pork quality is closely related to its intramuscular fat (IMF) content and chemical composition. The IMF contents have a positive correlation with tenderness, marbling score, and juiciness [4]. The IMF chemical composition mainly includes fatty acid contents and profiles that have an important effect on pork flavor and nutritional values, which is a key index for human health [5].

Therefore, the IMF regulation is the main strategy to improve pork quality. The IMF deposition and composition are regulated by many factors, mainly including animal breeds, diet composition, gender, etc. [4]. Numerous studies have shown that nutritional regulation is an efficient means of modulating IMF deposition and producing high-quality and healthy pork [6,7]. In recent years, probiotic microbial technology is a popular strategy to enhance growth performance and improve pork quality traits [8,9]. The fermented feed (FF) can degrade anti-nutritional factors, decompose organic compounds, enhance feed utilization efficiency, regulate intestinal microbiota balance, and improve the body’s immunity [10]. Therefore, the fermented feed had an important role in promoting the growth performance [8,11,12]. Additionally, microbial fermented feed can increase the content of flavor compounds in pork, thereby enhancing the taste flavor of pork [13]. The other studies have shown that the 10% microbial fermented feed markedly increased the contents of amino acids and polyunsaturated fatty acids (PUFAs) and monounsaturated fatty acids (MUFAs) that are flavor precursors [8]. Moreover, microbial fermented feed contributes to modulating gut microbial composition, promoting gut health, and improving meat quality [4,8]. The extensive research also clarified that the intestinal microorganisms had an important effect on IMF deposition and pork quality [1,14,15]. The multi-omics analysis also has demonstrated that gut microbiota and microbial metabolites (short-fatty acid and bile acids) have significant impact on lipid metabolism-related genes (*SREBP1c*, *PPARγ*, and *FAT*/*CD36*) and meat quality [8,14,16,17]. These findings suggested that the diet supplement with microbial fermented feed is an effective practice for producing high-quality pork.

Compared with Chinese indigenous fat-type pig breeds, Western lean-type pigs (mainly including Duroc, Landrace, and Yorkshire breeds) are preferred by producers due to their high lean rates, rapid growth, and efficient feed conversion rate. But the low intramuscular fat (IMF) content in lean-type pigs represents a key challenge, which compromises pork quality attributes, including tenderness, juiciness, and flavor [18,19]. Thus, this research mainly aimed to clarify the impacts of FF supplementation on the growth performance, pork quality characteristics, and pork nutritional values in the lean-type finishing pigs.

## 2. Materials and Methods

The research protocol was agreed by the Ethics Committee of Yangtze University (Jingzhou, China, approval No. DKYB20240302).

### 2.1. The Preparation of Microbial Fermented Feed

The microbial fermented feed was provided by a company (ChuTian AiKe, Qianjiang, China). Briefly, the fermentation substrate (20% corn, 25% wheat bran, and 55% soybean meal) was fermented by microbial consortium, including *Lactobacillus plantarum* (4.0 × 10^11^ CFU/g), *Bacillus coagulans* (3.0 × 10^11^ CFU/g), *Bacillus subtilis* (1.0 × 10^12^ CFU/g), and yeast (4.5 × 10^10^ CFU/g). The basal diet was processed to 30% moisture content and then inoculated with 4% of microbial combination liquid, and next, anaerobic fermentation was carried out at 24–34 °C for 3~4 days. The final ratio of solid to liquid was 2:1 (weight/volume). Then the fermented raw material was used to make the full feed, and the crude protein content of FF was determined (28.5%).

### 2.2. Animal Feeding and Management

A total of 80 finishing DLY (Duroc × Landrace ×Yorkshire) pigs (59 kg BW) were divided randomly into 2 dietary treatment groups, with 5 replications in each group and 8 pigs per replication in a pen. The pigs in the control group (CON) were fed with basal diet; the pigs in the fermented feed group (FF) were fed with basal diet supplementation 10% FF. The basal diet was formulated based on NRC (2012) guidelines of finishing pigs (Table 1). Pigs were fed ad libitum three times daily and had free access to water. The experiment lasted 70 days.

### 2.3. Sample Collection

After ending of feeding trial, 10 pigs randomly selected per treatment (2 pigs/replicate) were fasted for 12 h pre-slaughter, followed by intravenous blood sampling. Serum was isolated via centrifugation (3000× *g*, 10 min, 4 °C) and stored at −20 °C for biochemical and hormonal assay. Then the selected pigs were humanely slaughtered at a commercial facility using electrical stunning. At 45 min post-mortem, the average backfat thickness based on the first, tenth, and last ribs was measured with vernier caliper (Guangzhou, China). The *longissimus dorsi* muscle (LDM) samples of the carcasses were collected and frozen in liquid nitrogen and then stored at −80 °C for the analysis of fatty acid, muscle chemical composition, and gene expression analysis. The muscle samples for meat quality determination were stored at 4 °C. The colon digesta samples (about 2 g, 6 pigs per group) were collected in the sterile tube from the middle colon and frozen in liquid nitrogen and then stored at −80 °C for microbiota analysis.

### 2.4. Growth Performance Determination

The initial body weight and final body weight were recorded on the first and last day of the feeding trial to calculate the average daily gain (ADG). The feed consumption was recorded in whole trial to calculate the average daily feed intake (ADFI). And the feed-to-gain ratio (F/G) was determined by ADFI/ADG.

### 2.5. Serum Biochemical and Hormonal Assays

Serum biochemistry markers, including glucose (GLU), total cholesterol (TC), and triglycerides (TG), were analyzed by Mindray BS-420 automated analyzer, following the manufacturer’s guidelines (Shenzhen Mindray Bio-Medical Electronics Co., Ltd., Shenzhen, China). Additionally, serum hormone concentrations of insulin (H203-1-1, Nanjing Jiancheng Bioengineering Institute, Nanjing, China), leptin (H174-1-1, Nanjing Jiancheng Bioengineering Institute, Nanjing, China), and insulin-like growth factor 1 (IGF-1) (kt40315, Wuhan MSK Biotechnology Co., LTD, Wuhan, China) were determined by an ELISA methods according to the guidelines of the ELISA kit respectively.

### 2.6. Determination of Meat Quality and Meat Chemical Composition

The measurement methods of meat pH, drip loss, and shear force were presented in our previous study [20]. Briefly, the pH_45min_ and pH_24h_ were determined at 45 min and 24 h post-mortem, respectively, using portable AZ8694 pH meter (Taiwan Hengxin Co., Ltd., Shenzhen, China). The ΔpH was calculated as the differential value of pH_45min_ and pH_24h_. The shear force was detected by C-LM3 tenderness instrument (Xieli, Harbin, China) based on the manufacturer’s guidelines. Prior to the measurements, the samples’ packing vacuum bags were subjected to reheat at 80 °C water bath until the internal temperature reached 70 °C and cooled to room temperature. Before detecting the shear force, the samples were meticulously trimmed to uniform size with length 3 cm, width 2 cm, and thickness 1 cm, respectively.

To measure the drip loss, each LDM sample was trimmed to length 3 cm, width 2 cm, and thickness 1 cm forms at 24 h post-mortem. After weighting (recorded as W1), the sample packing in a plastic bag was suspended at 4 °C in the fridge for 24 h. Then the sample’s surface was gently dried using absorbent paper and weighted (recorded as W2). The drip loss was calculated by the formula: (W1 − W2)/W1 × 100%.

The meat color and marbling score were determined based on the method prior investigation [5]. Briefly, the meat color values (lightness L*, redness a*, and yellowness b*) were measured at 45 min and 24 h post-mortem, respectively, using CR-410 colorimeter (Konica Minolta, Osaka, Japan). Then the difference (∆E) was calculated as following formula [5]:

$$\Delta Ε=\sqrt{{\left(\Delta L^{*}\right)}^{2}+{\left(\Delta a^{*}\right)}^{2}+{\left(\Delta b^{*}\right)}^{2}}$$

The marbling score was evaluated according to the NPPC color chart (Mingao, Nanjing, China) at 45 min post-mortem.

The muscle contents of water, crude fat, crude protein, and crude ash were detected according to AOAC (2000) official guideline methods.

### 2.7. Fatty Acid Composition and Nutritional Value

The fatty acid contents in LDM were measured using gas chromatography instrument (Aglient7890, Agilent, Palo Alto, CA, USA) as described previously [1]. Briefly, the LDM sample was vacuum-dried using a freeze dryer and ground into a powder for analysis with a gas chromatograph. A quantitative sample was weighed and transferred to a 10 mL tube. Then the sample was extracted using benzene and petroleum ether mixture (2:1; *v*/*v*) overnight in the dark. After adding 2 mL KOH/methanol solution (4 mol/L) into the tube, the mixture solution was adjusted to volume 10 mL with pure water. Then the mixture solution was centrifuged, and the 100 µL supernatant was collected for fatty acid content determination using the gas chromatograph.

In present study, the determination of fatty acid included the saturated fatty acids (SFAs, mainly including C10:0, C12:0, C14:0, C16:0, C18:0, and C20:0), monounsaturated fatty acids (MUFAs, mainly including C16:1, C18:1n-9c, and C20:1n-9), and polyunsaturated fatty acids (PUFAs, mainly including C18:2n-6, C18:3n6, C18:3n-3, C20:4n-6, C20:5n-3, and C22:6n-3). The PUFAs included n-6 PUFAs (C18:2n-6, C18:3n-6, and C20:4n-6) and total n-3 PUFAs (C18:3n-3, C20:5n3, and C22:6n-3). DHA is docosahexaenoic acid (C22:6n-3), and EPA is eicosapentaenoic acid (C20:5 n-3). Based on the previous research [6,21], the indicators of the fatty acids nutritional value were fatty acids unsaturation index (UI), peroxidation trend index (PI), nutrition value index (NVI), index of atherogenicity (IA), index of thrombogenicity (IT), hypocholesterolemic-to-hypercholesterolemic ratio (HH ratio), and health-promoting index (HPI). The calculation formula for each index is as follows:

UI = (1 × monoenoic acid percent) + (2 × dienoic percent) + (3 × trienoic percent) + (4 × tetraenoic percent) + (5 × pentaenoic percent) + (6 × hexaenoic percentage)

PI = (0.025 × monoenoic acid percent) + (1 × dienoyl acid percent) + (2 × trienoic acid percent) + (4 × tetraenoic acid percent) + (6 × pentaenoic acid percent) + (8 × hexanoic acid percent)

NVI = (C18:0 percent + C18:1n-9 percent)/(C16:0 percent);

HPI = (ΣUFA)/(4 × C14:0 percent + C16:0 percent)

IA = (4 × C14:0 percent + C16:0 percent)/(ΣMUFA + ΣPUFA)

IT = (C14:0 percent + C16:0 percent + C18:0 percent)/[(0.5 × MUFA percent) + (0.5 × Σn-6PUFA percent) + (3 × Σn-3PUFA percent) + (Σn-3PUFA/Σn-6PUFA)]

HH ratio = (C18:1 percent + ΣPUFA)/(C14:0 percent + C16:0 percent)

HPI = (ΣUFA)/(4 × C14:0 percent + C16:0 percent)

### 2.8. Real-Time Quantitative PCR Analysis

Methods of RNA extraction and qRT-PCR of the LDM samples were followed from our previous research [22]. Briefly, RNA was extracted by using Trizol methods. Then the first-strand cDNA synthesis was conducted using FastKing cDNA First-strand synthesis kit (Beijing Tiangen Biotechnology Co., Ltd.Beijing, China). The real-time quantitative PCR analysis was performed by using fluorescence quantitative PCR kit (Sigma Aldrich, MO, USA). The primers sequence (Table 2) was designed using the Primer 5.0 software. The β-actin gene was used as an internal gene. And the 2^−ΔΔCT^ method was used to calculate the relative mRNA expression.

### 2.9. DNA Extraction and Sequencing of 16S rDNA

The colon 16S rDNA sequencing was performed as we previously reported [22]. In short, the colon sample DNA was extracted using DNA extraction kit (Omega, Norcross, GA, USA). And DNA concentration and purity were measured using micro fluorometer and spectrophotometers (ThermoScientific, Waltham, MA, USA), respectively. The V3-V4 regions of the bacterial 16S rRNA gene were amplified using universal primers (338F, 5′-TACGGGNGGCGCAG-3′; 806R, 5′-GATCACHVGGGTATCTAATCC-3′) by the PCR system (ABI GeneAmp 9700, Foster, CA, USA). Then the PCR products were purified using DNA gel extraction kit (Axygen, Union City, CA, USA) and quantified using the QuantiFluor^TM^ fluorometer (Promega, Madison, WI, USA). Finally, the purified PCR products were subjected to DNA sequencing of paired-end on a sequencing system (Illumina MiSeq platform, San Diego, CA, USA) according to standard protocols (Majorbio Bio-Pharm Technology Co., Ltd., Shanghai, China).

The bioinformatics process mainly included data filtering, DADA2 denoising, taxonomic assignment, etc. The resulting sequences were quality-filtered based on fastp (0.19.6) and merged with FLASH (v1.2.11). Then the high-quality sequences were denoised using the DADA2 plugin in the Qiime2 (version 2020.2) pipeline with recommended parameters to obtain amplicon sequence variants (ASVs) sequence. Taxonomic assignment of ASVs was performed using the Naïve Bayes consensus taxonomy classifier implemented in Qiime2 and the SILVA 16S rRNA database. The data were analyzed by Majorbio Cloud Platform (https://www.majorbio.com/).

### 2.10. Statistical Analysis

The SPSS 22 software was used for statistical analyses. Experimental data were presented as the means ± SE (standard error). Means were compared by Student’s *t*-test. *p* < 0.05 was considered a significant difference.

## 3. Results

### 3.1. Growth Performance 

The growth performance was shown in Table 3. Compared with control group, fermented feed significantly increased final body weight and ADG (*p* < 0.05) while significantly decreased the F/G (*p* < 0.05), showing a significant effect on ADG and F/G.

### 3.2. Serum Biochemical and Hormonal Analysis

The levels of the lipid metabolism-related biochemical parameters and hormones were presented in Table 4. The levels of serum total cholesterol, triglyceride, insulin, leptin, and IGF-1 in FF group were significantly higher than in CON group (*p* < 0.05), while the glucose level did not significantly differ between groups (*p* > 0.05).

### 3.3. Meat Quality

The meat quality indices were presented in Table 5. There were no significant differences in pH_45min_, pH_24h_, and backfat thickness between two groups. However, the ΔpH in FF treatment was significantly lower than that in CON treatment (*p* < 0.05). Compared with CON group, the fermented feed significantly reduced the color values of L*_45min_, b*_45min_, L*_24h_, and b*_24h_ and increased the color values of a*_45min_, a*_24h_, and ΔE (45 min–24 h) (*p* < 0.05). The drip loss and shear force of pigs in CON group were significantly lower, and marbling scores were higher than those of the pigs in FF group (*p* < 0.05).

### 3.4. Chemical Composition and Lipid Metabolism-Related RNA Expression

The LDM chemical compositions were presented in Table 6. The LDM IMF content was significantly elevated in FF group compared to CON group (*p* < 0.05), while the contents of moisture, crude protein, and crude ash in LDM showed no significant differences between two dietary treatments.

**Figure 1:**
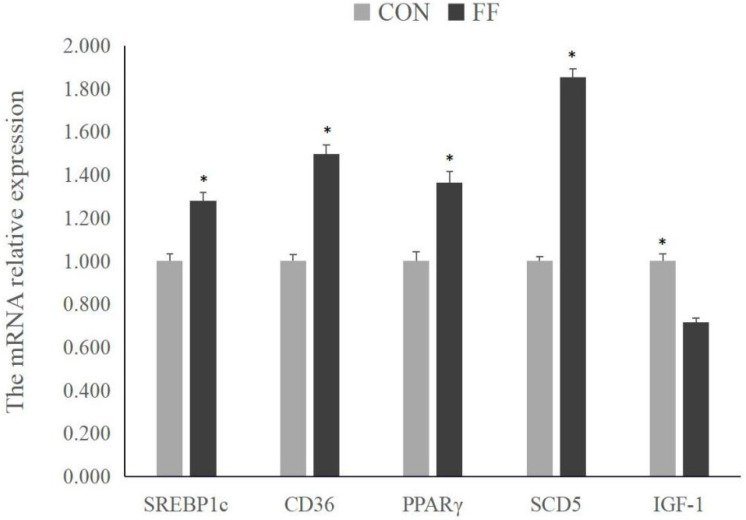
The lipid-related gene expression. Note: CON = control group (basal diet); FF = fermented feed group; IGF-1 = insulin-like growth factor-1; SREBP1c = sterol regulatory element binding protein 1c; PPARγ = peroxisome proliferator-activated receptor γ; SCD5 = stearoyl-CoA desaturated enzyme 5; FAT/CD36 = fatty acid translocase/CD36. * Significantly different compared with the control group at *p* < 0.05.

The gene expression-related fatty acid uptake and transport (*FAT*/*CD36*), fatty acid synthesis (*SREBP1c*, *PPARγ*, and *SCD5*), and lipid metabolism (IGF-1) were presented in Figure 1. The fermented feed significantly promoted the expression of *SREBP1c*, *PPARγ*, *SCD5*, and *FAT/CD36* mRNA (*p* < 0.05) and decreased the *IGF-1* mRNA levels (*p* < 0.05).

### 3.5. Muscle Fatty Acid Profiles and Nutritional Values

The FF had an important effect on the LDM fatty acid content (Table 7). Compared with CON group, FF supplementation markedly elevated levels of total MUFAs (especially C18:1 n-9) and total PUFAs (especially C18:2 n-6 and C20:4 n-6) in LDM (*p* < 0.05) while significantly reducing the saturated fatty acids C18:0 contents (*p* < 0.05).

The evaluation of fatty acids nutritional values (Table 8) indicated that the fermented feed significantly elevated the indices of PUFA:SFA, UI, and PI compared to CON group and significantly reduced the IA value (*p* < 0.05). The indices of DHA + EPA, NVI, HPI, IT, and HH ratio were exhibited in non-difference between two dietary groups (*p* > 0.05).

**Table 8:** Effects of FF on the nutritional value indexes of the LDM in finishing pigs.

Item	Treatment		*p*-Value
	CON	FF	
DHA + EPA	0.19 ± 0.02	0.17 ± 0.03	0.315
PUFAs: SFA	0.43 ± 0.01 ^b^	0.48 ± 0.01 ^a^	0.024
UI	74.48 ± 3.36 ^b^	79.93 ± 2.45 ^a^	0.032
PI	21.86 ± 1.13 ^b^	23.84 ± 1.17 ^a^	0.027
NVI	2.21 ± 0.15	2.29 ± 0.13	0.513
HPI	2.02 ± 0.14	2.19 ± 0.15	0.264
IA	0.495 ± 0.02 ^a^	0.457 ± 0.02 ^b^	0.018
IT	1.22 ± 0.02	1.12 ± 0.01	0.235
HH ratio	2.22 ± 0.03	2.4 ± 0.02	0.061

^a,b^ Data with different superscript letters are significantly different (*p* < 0.05). Note: Data are expressed as the means ± SE (standard error), n = 10. CON = control group (basal diet); FF = fermented feed group; DHA = docosahexaenoic acid, C22:6 n-3; EPA = eicosapentaenoic acid, C20:5 n-3; PUFAs = polyunsaturated fatty acids; SFA = saturated fatty acid; UI = fatty acids unsaturation index; PI = peroxidation trend index; NVI = nutrition value index; HPI = health-promoting index; IA = index of atherogenicity; IT = index of thrombogenicity; HH ratio = hypocholesterolemic-to-hypercholesterolemic ratio.

### 3.6. Colonic Microbiota Communities

**Figure 2:**
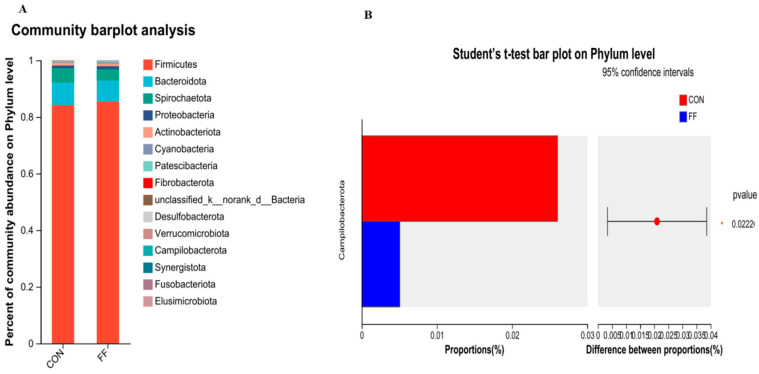
Relative abundance of colonic microbiota at phylum level. Note: CON = control group (basal diet); FF = fermented feed group, n = 6. (**A**) Relative abundance of colonic microbiota phylum for CON and FF groups ^(1)^. (**B**) The different abundance of microbiota at phylum level between CON and FF groups by *t*-test analysis.

**Figure 3:**
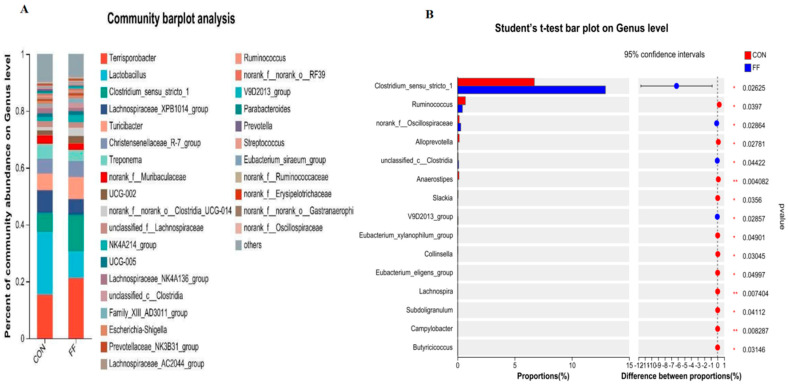
Relative abundance of colonic microbiota at genus level. Note: CON = control group (basal diet); FF = fermented feed group, n = 6. (**A**) Relative abundance of colonic microbiota genus for CON and FF group. (**B**) The different abundance of microbiota at genus level between CON and FF groups by *t*-test analysis.

**Figure 4:**
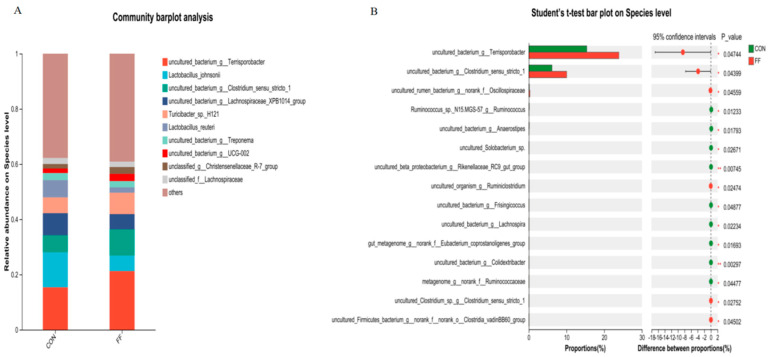
Relative abundance of colonic microbiota at species level. Note: CON = control group (basal diet); FF = fermented feed group, n = 6. (**A**) Relative abundance of colonic microbiota species for CON and FF groups. (**B**) The different abundance of microbiota at species level between CON and FF groups by *t*-test analysis.

The colonic microbiota communities were analyzed in Figure 2 and Figure 3. The most abundant phyla were presented in Figure 2A. The dominant phyla composition was Bacteroidetes, Firmicutes, Spirochaetota, and Proteobacteria in the colon microbiota of two groups. Compared with the control, fermented feed significantly reduced the *Campilobacterota* abundance at the phylum level (Figure 2B, *p* < 0.05). The top 30 wealthiest genera in colon were presented in Figure 3A at the genus level. Compared with CON group, the genus abundances of *Clostridium_sensu_stricto*, *norank_f_Oscillospiraceae*, *unclassified_c_Clostridia*, and *V9D2013* were significantly elevated in FF group compared to CON group (Figure 3B, *p* < 0.05). The top 20 wealthiest species in colon were presented in Figure 4A at the species level. Compared with CON group, the species abundances of *g__Terrisporobacter*, *g__Clostridium_sensu_stricto_1*, *g__norank_f__Oscillospiraceae*, *g__Ruminiclostridium*, and *Clostridia_vadinBB60_group* were significantly elevated in FF group compared to CON group (Figure 4B, *p* < 0.05).

## 4. Discussion

Meat quality is a vital factor in the economics of pig production. Though the Western commercial lean-type pig breeds tend to have higher lean meat yield and better growth performance than Chinese indigenous fat-type pig breeds, lean-type pigs exhibit less meat quality, mainly including meat tenderness, taste flavor, and color [23,24]. Therefore, it is an important objective to produce high-quality pork in lean-type pigs production. Nutritional regulation has been verified to be an effective approach to promote pork quality. The extensive research has established that microbial fermented feed can elevate pork quality and boost consumer’s acceptability [8,9,25]. In China, the major components of pig diets are corn and soybean meal. The corn and soybean meal fermented by prebiotic compounds of *Bacillus coagulans*, *Lactobacillus plantarum*, and *Bacillus subtilis* not only reduced the lower anti-nutritional factors levels, but also exhibited effective modulation on growth traits and meat quality [26,27]. The research showed that yeast-fermented feed markedly enhanced the milk quality and improved the milk fatty acid profiles of ruminants [28]. Therefore, this research utilized the multi-strain (*Bacillus coagulans*, *Lactobacillus plantarum*, *Bacillus subtilis,* and yeast) to ferment corn and soybean meal diet and to enhance the regulation effects on growth performance traits and meat quality. Moreover, the dosage of strains in fermentation referenced the previous studies [8,11,29]. The results also showed that the specific multi-strain fermented feed significantly increased the marbling scores, improved the tenderness of pork, and decreased the drip loss of pork, in line with previous reports [12,30]. This finding showed that mixed-microbial fermented feed supplementation exhibited the effective strategy to modulate meat quality and nutritional values. Though mixed-microbial fermented feed supplementation had an efficient regulation on growth performance, meat quality, immunity function, intestinal health, and animal welfare [8,11,12,29], it is very difficult to quantify and identify the different strains’ microorganisms in the diet supplementation fermented feed [31,32]. Especially, it is very hard to quantify and identify *Bacillus coagulans* and *Bacillus subtilis* in the diet. Up to now, the articles in this area are very scarce.

The mechanism of meat quality traits formation is the results of a series of biochemical reaction in the muscle after slaughter. Following the slaughter, pork typically undergoes muscle glycolysis metabolism, which results in a decrease in pH value. Meat pH value is a critical indicator of meat quality because the pH is closely linked to meat color, drip loss, and shear force [33,34]. When muscle pH declines to the isoelectric point, muscle proteins undergo the strongest dehydration, which leads to lower water-holding capacity. Furthermore, water loss is accompanied by the change of the meat color, exhibiting paleness [35]. Therefore, the higher ΔpH may result in higher meat drip loss and poor color [34]. The present study also confirmed that the lower ΔpH with fermented feed supplementation is to be accompanied by lower drip loss and higher ΔE.

Numerous studies showed that IMF content is an important meat quality index, which affects the many meat quality traits, such as taste flavor, tenderness, and nutritional values [17,23,24]. The lipid deposition in muscle is modulated by several lipid metabolism-related genes, such as *PPARγ*, *SREBP1c*, *SCD5*, and *FAT*/*CD36*. PPARγ is a major regulator for fatty acid uptake and triglyceride formation in adipose cells [36]. SREBP-1c involves glucose utilization and fatty acid synthesis. FAT/CD36 is a fatty acid transporter participating in muscle lipid metabolism and promoting fatty acid uptake [37]. SCD5 cascade plays a critical role in regulating fatty acid desaturation and, therefore, promoting the synthesis of PUFA [37]. The upregulation of these fatty acid metabolism-related gene expression was associated with higher IMF contents and better taste flavor [38]. This study also revealed that fermented feed promoted the expression of *PPARγ*, *SREBP1c*, *SCD5*, and *FAT/CD36* genes and increased the IMF contents in LDM, suggesting that microbial fermented feed may be an effective strategy to regulate lipid metabolism of lean-type pigs. Our findings were also similar to other studies, which indicated that FF significantly increased the IMF contents and improved the pork quality [8].

The types and contents of fatty acids in pork have an important effect on the flavor quality and nutritional values [39]. Fatty acids include SFAs, MUFAs, and PUFAs. PUFA (e.g., oleic acid, docosahexaenoic acid, and alinolenic acid) is an important precursor for meat flavor [40], which can react with esters, ketones, and alcohols under heating to produce meat flavor [41]. Previous studies have shown that some neutral lipid fatty acids, such as C18:2 n-6 and C22:5 n-3, had a negative correlation with pork flavor and acceptability, whereas other fatty acids, such as C16:1 and C18:1, had a positive correlation with pork flavor, overall acceptability, and human health [42]. Moreover, the PUFA contents and profiles also affect the pork nutritional values. PUFA/SFA, UI, PI, and IA are confirmed to be critical indicators of meat fatty acids nutritional values [6,43]. The polyunsaturated fatty acids (PUFAs) exhibit anti-atherogenic properties; therefore, a higher PUFA:SFA ratio is more beneficial to cardiovascular health. The UI index mainly evaluates fatty acid unsaturation degree and high-quality PUFA. The PI index shows the effect of FAs on coronary artery disease. The IA index indicates the atherogenic and thrombogenic potential of FAs. It is believed that higher UI and PI indices and lower IA index might enhance pork meat nutritional value and health benefits [6,43]. Fermented mixed feed significantly raised the contents of oleic acid (C18:1), linoleic acid (C18:2), arachidonic acid (C20:4), and total UFA, but decreased total SFA in pork [25]. Especially, yeast-fermented feed exhibited good regulation on milk quality and milk fatty acid profiles of ruminants [28]. And MUFAs (such as C18:1) are associated with desirable meat quality traits [35]. Our results also revealed that FF decreased significantly the contents of C18:0 and total SFA and significantly increased the levels of C18:1 n-9, C18:2 n-6 and C20:4 n-6, total MUFA, and total PUFA in pork. Meanwhile, the present research also revealed that fermented feed elevated the PUFA/SFA, UI, and PI indices and decreased the IA index. These results indicated that microbial fermented feed can effectively promote pork nutritional values.

The numerous studies have shown that gut microbiota have prominent effects on meat quality [8,13,15]. Notably, meat quality, including meat color, drip loss, and tenderness, is closely related to antioxidant status. Studies have shown that diet-supplemented antioxidants can change composition of gut microbiota and reduce oxidative stress, thereby improving the meat quality [15]. In this study, *Lactobacillus plantarum*, *Bacillus coagulans*, *Bacillus subtilis*, and yeast were chosen as the starting strains. Among them, the cell wall and cell contents of yeast have multiple effects on improving antioxidant and immune functions in animals. Furthermore, previous reports have shown that probiotic fermentation improved the level of antioxidants (e.g., SOD, CAT, and GSH-Px) in feed [44]. Therefore, FF may improve the antioxidant function of feed through microbial metabolites and then improve the antioxidant level of the animal muscle, improving meat color while decreasing drip loss.

The regulation of gut microbiota on meat quality may take part in the lipid metabolism of the host [17,45,46]. The studies indicated that the deposition of IMF was closely associated with gut microbial composition, diversity, abundance, and metabolites, mainly including short-chain fatty acids (SCFAs) and bile acids [1,47]. Many reports have also demonstrated the regulatory roles on lipid metabolism of certain probiotics, including *Lactobacillus* and *Bifidobacterium* [48]. In this study, we showed that the abundance of *Clostridium* was markedly increased by FF. A previous study also uncovered that increased *Clostridium* abundance and the firmicutes-to-bacteroidetes ratio were positively correlated with IMF [25], which was consistent with our data. Xie et al. also identified the key four microbiota species (including *Bacteroides uniformis*, *Sphaerochaeta globosa*, *Hydrogenoanaerobacterium saccharovorans*, and *Pyramidobacter piscolens*) for regulating lipid metabolism and deposition [17]. The research also showed that gut microbes may participate in the regulation of lipid metabolism through PPARγ signaling pathway [37]. PPARγ takes part in fatty acid uptake, triglyceride formation, and storage in lipid droplets [36]. This study showed that fermented feed significantly increased the *Clostridium* abundance at the genus level. The increased *Clostridium* could promote the secondary bile acid biosynthesis through regulating *Clostridium*-mediated 7αdehydroxylation activity, which suppress FXR receptor signaling and activate *PPARγ* and *SREBPC* expression [48,49]. But genus *Clostridium* comprises approximately 180 species, which can exhibit beneficial or harmful effects in animals [50]. In this study, the fermented feed significantly increased the abundance of the *g_Clostridium_sensu_stricto_1* species, *Clostridia_vadinBB60_group* species, and *Terrisporobacter* species. Some research has reported that these species had a beneficial function for pigs [51,52]. Niu et al. (2022) reported that *Clostridium_sensu_stricto_1* and *Terrisporobacter* were closely associated with fatty acid contents in LDM [53], which may be an important reason for fermented feed regulating pork quality and fatty acid profiles.

## 5. Conclusions

Enhancing pork quality and flavor has always been a major concern for lean-type pig producers. The current study revealed that the diet supplementation with 10% fermented feed significantly improved growth traits, pork quality, IMF contents, IMF deposition-related gene expression, and fatty acid nutritional values, which provided strong evidence for the extensive application of microbial fermented feed. Taken together, the novelty of this study is to provide the nutritional regulative strategy to improve meat quality of lean-type pig breeds. However, the application of microbial fermented feed is a complex technology, and the fermentation process, strain selection, and microbial dosage will affect the effect of fermented feed, which should draw attention in future research.

## Figures and Tables

**Table 1:** Ingredients and nutrient levels of diets.

Item	CON	FF
Corn, %	63.0	51.0
Soybean meal, %	23.5	26.0
Wheat bran, %	9.0	9.5
Fermented feed, %	0	10.0
Premix ^(^^1)^, %	4.0	4.0
Total	100.0	100.0
Nutrient level ^(2)^		
Digestible energy, MJ/kg	13.2	13.3
Crude protein, %	16.5	16.5
Lysine, %	0.90	0.92
Calcium, %	0.75	0.78
Available phosphorus, %	0.32	0.33

Note: CON = control group (basal diet); FF = fermented feed group. ^(1)^ Provided the following per kilogram of complete diet: vitamin A, 5000 IU; vitamin D3, 1200 IU; vitamin E, 54 IU; vitamin B1, 1.8 mg; vitamin B2, 3.5 mg; vitamin B12, 0.02 mg; vitamin B6, 3 mg; D-pantothenic acid, 24 mg; nicotinic acid, 25 mg; Cu, 10 mg; Fe, 55 mg; Zn, 80 mg; Mn, 30 mg; I, 0.5 mg; Se, 0.25 mg; Co, 0.012 mg. ^(2)^ Digestible energy was shown as a calculated value. The other nutrient levels were obtained through actual measurements.

**Table 2:** The primers of qPCR.

Gene	Primer	Size/bp	Gene Bank ID
*β-actin*	Forward: CCACGAAACTACCTTCAACTC Reverse: TGATCTCCTTCTGCATCCTGT	131	NM_414396
*IGF-1*	Forward: AGCCCACAGCCTACGCCTC Reverse: CTTCTGAGCCTTGGCCATCTC	179	NM_101055488
*SREBP1c*	Forward: GCGACGGTGCCTCTGGTAGT Reverse: CGCAAGACGGCGGATTTA	218	NM_214157.1
*PPARγ*	Forward: CTGACCAAAGCAAAG-GCG Reverse: TGGCG- TAGAGGTCCTTGCG	162	NM_214379.1
*SCD5*	Forward: GCCACCTTTCTTCGTTACG Reverse: CCTCACCCACAGCTCCCAAT	142	NM_125137883
*FAT/CD36*	Forward: ACCCTGAGACCCCACACAGTC Reverse: TACAGCTGCCACAGCCAGAT	156	NM_733702

Note: IGF-1 = insulin-like growth factor-1; SREBP1c = sterol regulatory element binding protein 1c; PPARγ = peroxisome proliferator-activated receptor γ; SCD5 = stearoyl-CoA desaturated enzyme 5; FAT/CD36 = fatty acid translocase/CD36.

**Table 3:** Effects of FF on the growth performance of finishing pigs.

Item	Treatment		*p*-Value
	CON	FF	
Initial body weight, kg	59.19 ± 2.32	59.34 ± 1.27	0.827
Final body weight, kg	112.53 ± 2.41	117.64 ± 2.53	0.035
ADFI, kg	2.37 ± 0.02	2.38 ± 0.02	0.862
ADG, kg	0.762 ± 0.01 ^b^	0.819 ± 0.02 ^a^	0.027
F/G	3.11 ± 0.03	91 ± 0.05	0.012

^a,b^ Data with different superscript letters are significantly different (*p* < 0.05). Note: Data are expressed as the means ± SE (standard error), n = 40. CON = control group (basal diet); FF = fermented feed group; ADG = average daily gain; ADFI = average daily feed intake; F/G = feed-to-gain ratio.

**Table 4:** Effects of FF on the serum biochemical and hormonal parameters of finishing pigs.

Item	Treatment		*p*-Value
	CON	FF	
Glucose (mmol/L)	5.12 ± 1.21	5.47 ± 1.18	0.174
Total cholesterol (mmol/L)	2.24 ± 0.13 ^b^	2.85 ± 0.11 ^a^	0.042
Triglyceride (mmol/L)	0.61 ± 0.03	0.75 ± 0.04	0.093
Insulin (ng/mL)	25.15 ± 5.18 ^b^	28.92 ± 6.21 ^a^	0.008
Leptin (ng/mL)	31.21 ± 2.13 ^b^	41.52 ± 2.17 ^a^	0.036
IGF-1 (ng/mL)	148 ± 9.26 ^b^	162 ± 8.69 ^a^	0.018

^a,b^ Data with different superscript letters are significantly different (*p* < 0.05). Note: Data are expressed as the means ± SE (standard error), n = 10. CON = control group (basal diet); FF = fermented feed group; IGF-1 = insulin-like growth factor.

**Table 5:** Effects of FF on the meat quality of finishing pigs.

Item	Treatment		*p*-Value
	CON	FF	
pH_45min_	6.47 ± 0.06	6.38 ± 0.05	0.585
pH_24h_	5.66 ± 0.04	5.73 ± 0.05	0.157
ΔpH	0.81 ± 0.03 ^a^	0.65 ± 0.03 ^b^	0.025
Lightness, L*_45min_	45.62 ± 1.32 ^a^	41.63 ± 0.28 ^b^	0.026
Redness, a*_45min_	11.48 ± 0.35 ^b^	12.16 ± 0.42 ^a^	0.015
Yellowness, b*_45min_	8.74 ± 0.17 ^a^	8.16 ± 0.21 ^b^	0.034
Lightness, L*_24h_	47.25 ± 1.69 ^a^	43.18 ± 1.38 ^b^	0.006
Redness, a*_24h_	13.17 ± 0.17 ^b^	14.65 ± 0.14 ^a^	0.014
Yellowness, b*_24h_	9.36 ± 0.16 ^a^	8.32 ± 0.15 ^b^	0.032
ΔE (45 min–24 h)	2.43 ± 0.09 ^b^	2.94 ± 0.13 ^a^	0.015
Drip loss, %	1.35 ± 0.07 ^a^	1.22 ± 0.06 ^b^	0.026
Shear force, kg.f	2.40 ± 0.02 ^a^	2.05 ± 0.03 ^b^	0.022
Marbling scores	3.3 ± 0.06 ^b^	3.5 ± 0.05 ^a^	0.017
Backfat thickness, mm	3.08 ± 0.11	3.14 ± 0.13	0.082

^a,b^ Data with different superscript letters are significantly different (*p* < 0.05). Note: Data are expressed as the means ± SE (standard error), n = 10. CON = control group (basal diet); FF = fermented feed group.

**Table 6:** Effects of FF on the LDM chemical composition of finishing pigs (%, as-fresh basis).

Treatment	Moisture	Crude Protein	IMF	Crude Ash
CON	70.30 ± 0.01	21.41 ± 0.01	3.36 ± 0.01 ^b^	3.54 ± 0.02
FF	69.86 ± 0.01	21.64 ± 0.01	3.79 ± 0.01 ^a^	3.42 ± 0.01
*p*-value	0.592	0.724	0.005	0.862

^a,b^ Data with different superscript letters are significantly different (*p* < 0.05). Note: Data are expressed as the means ± SE (standard error), n = 10. CON = control group (basal diet); FF = fermented feed group; IMF = intramuscular fat.

**Table 7:** Effects of FF on the FA contents of the LDM in finishing pigs (%, as-fresh basis).

Item	Treatment		*p*-Value
	CON	FF	
C10:0	0.32 ± 0.01	0.26 ± 0.02	0.174
C12:0	0.063 ± 0.002	0.072 ± 0.001	0.142
C14:0	1.31 ± 0.031	1.25 ± 0.048	0.093
C16:0	22.15 ± 0.18	21.92 ± 0.16	0.108
C18:0	12.21 ± 0.13 ^a^	11.52 ± 0.17 ^b^	0.036
C20:0	0.24 ± 0.01	0.25 ± 0.01	0.418
C16:1	2.91 ± 0.03	2.95 ± 0.05	0.237
C18:1n-9	36.66 ± 1.69 ^b^	38.78 ± 1.54 ^a^	0.006
C20:1	0.22 ± 0.001	0.23 ± 0.003	0.281
C18:2n-6	13.53 ± 1.27 ^b^	14.85 ± 1.61 ^a^	0.026
C18:3n-3	0.46 ± 0.01	0.45 ± 0.01	0.215
C18:3n-6	0.15 ± 0.07	0.17 ± 0.03	0.127
C20:4n-6	1.18 ± 0.03 ^b^	1.36 ± 0.08 ^a^	0.017
C20:5n-3	0.06 ± 0.01	0.05 ± 0.01	0.309
C22:6n-3	0.13 ± 0.02	0.12 ± 0.03	0.083
Total saturated fatty acids	36.29 ± 1.58 ^a^	35.27 ± 1.73 ^b^	0.035
Total monounsaturated fatty acids	39.79 ± 1.75 ^b^	41.96 ± 2.16 ^a^	0.015
Total polyunsaturated fatty acids	15.51 ± 1.36 ^b^	17.00 ± 1.52 ^a^	0.031

^a,b^ Data with different superscript letters are significantly different (*p* < 0.05). Note: Data are expressed as the means ± SE (standard error), n = 10. CON = control group (basal diet); FF = fermented feed group.

## Data Availability

The original contributions presented in this study are included in the article; further inquiries can be directed to the corresponding authors.
